# Identification and evaluation of semiochemicals for the biological control of the beetle *Omorgus suberosus* (F.) (Coleoptera: Trogidae), a facultative predator of eggs of the sea turtle *Lepidochelys olivacea* (Eschscholtz)

**DOI:** 10.1371/journal.pone.0172015

**Published:** 2017-02-13

**Authors:** Vieyle Cortez, José R. Verdú, Antonio J. Ortiz, Gonzalo Halffter

**Affiliations:** 1 I.U.I. CIBIO, Universidad de Alicante, Alicante, Spain; 2 Departamento de Química Inorgánica y Química Orgánica, EPS Linares, Universidad de Jaén, Linares, Spain; 3 Instituto de Ecología, A C, Red de Ecoetología, Xalapa, Veracruz, México; Universidade Federal de Vicosa, BRAZIL

## Abstract

The beetle *Omorgus suberosus* (F.) is a facultative predator of eggs of the olive ridley turtle *Lepidochelys olivacea* (Eschscholtz). Laboratory and field investigations were conducted in order to characterize volatile attractants of *O*. *suberosus* and to explore the potential for application of these volatiles in a selective mass trapping method. Headspace sorptive extraction (HSSE) coupled to thermo-desorption gas chromatography–mass spectrometry (TD-GC-MS) analysis of the volatile constituents from beetles or turtle nests revealed 24 potential compounds. However, electroantennographic (EAG) measurements revealed antennal sensitivity only to indole, linoleic acid, trimethylamine, dimethyl sulphide, dimethyl disulphide and ammonia. Behavioural tests showed that these compounds are highly attractive to *O*. *suberosus*. Field trapping experiments revealed that indole and ammonia were more attractive than the other volatile compounds and showed similar attractiveness to that produced by conventional baits (chicken feathers). The use of a combined bait of indole and NH_3_ would therefore be the most effective trap design. The data presented are the first to demonstrate effective massive capture of *O*. *suberosus* using an attractant-based trapping method. These findings have potential for the development of an efficient mass trapping method for control of this beetle as part of efforts towards conservation of *L*. *olivacea* at La Escobilla in Oaxaca, Mexico.

## Introduction

**Fig 1:**
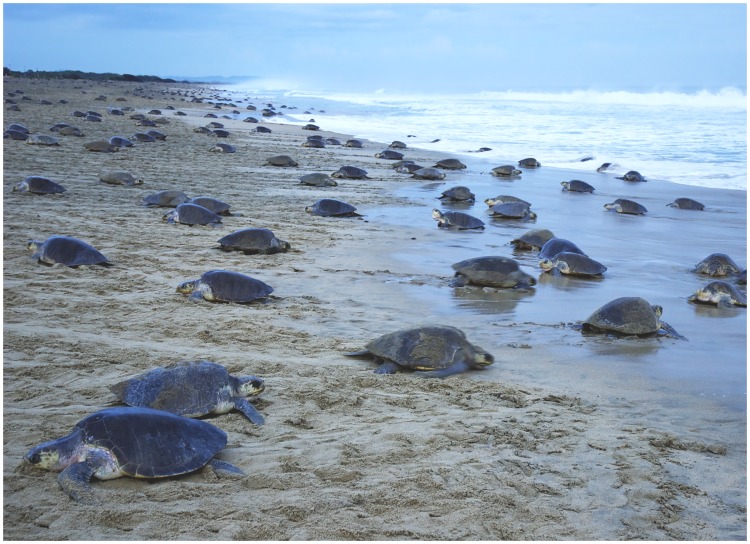
Synchronous nesting event (‘arribada’) of *Lepidochelys olivacea* in the Natural Sanctuary ‘La Escobilla’ (Oaxaca, Mexico).

**Fig 2:**
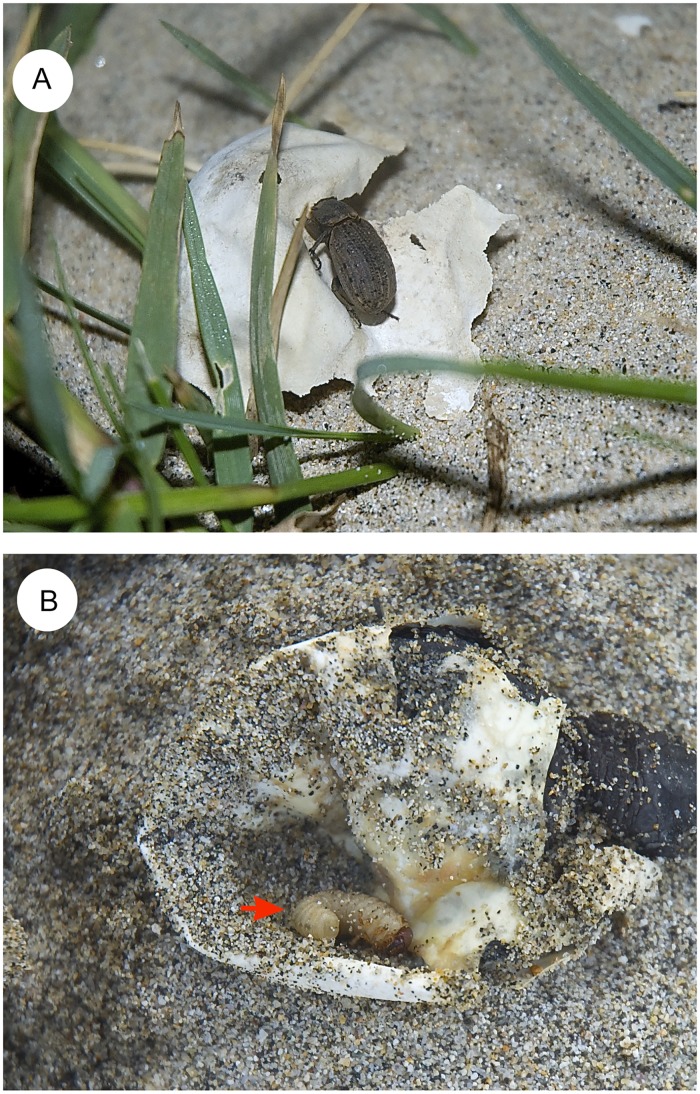
Feeding behaviour of *Omorgus suberosus* in the Natural Sanctuary ‘La Escobilla’ (Oaxaca, Mexico). Necro–saprophagous behaviour of adult *Omorgus suberosus* (A) on the *Lepidochelys olivacea* egg shell, and predation of turtle eggs by *O*. *suberosus* larvae (marked by arrow).

The olive ridley turtle *Lepidochelys olivacea* (Eschscholtz) is listed as ‘vulnerable’ by IUCN [1] and classified as ‘endangered’ in the Mexican national red list (NOM-059-SEMARNAT-2010). This sea turtle is a pantropical marine species, distributed in tropical and subtropical regions of the oceans worldwide. The olive ridley turtle is particularly known for its mass synchronous nesting events, or ‘arribadas’ (Spanish for arrivals), a common nesting phenomenon in the eastern Pacific, western Atlantic and northern Indian Oceans [2–9]. One of the most important nesting areas of this turtle is La Escobilla Beach in the state of Oaxaca on the Mexican coasts of the eastern Pacific, where more than one million turtles arrive every year [2,9,10] (Fig 1). The Escobilla sea turtle sanctuary was created in 1986 to protect the olive ridley turtle, under the responsibility of National Commission of Protected Natural Areas (CONANP). This is a protected natural area with the category of sanctuary (Category IV, CONANP). It has recently been observed that the high density of olive ridley turtle nests on this beach represents a seasonal food source for the opportunist beetle *Omorgus suberosus* (F.) (Coleoptera: Scarabaeoidea: Trogidae). The high abundance of this beetle recorded on this beach over the last fifteen years is of great concern, since this opportunistic attack is considered a risk to the survival of this turtle species. Based on historical data, a dramatic decrease in the olive ridley turtle populations has been reported for all historical ‘arribada rookeries’ sites (–99.5%, during two generations or its equivalent of 40 years for this species) with exception of the La Escobilla, in which a notable increase about 126% has been recorded in the same period [1,2]. In terms of number of individuals, this increase represents more than 10^6^ females nesting in no more than 7 km of coastline. Some hypotheses could explain this phenomenon but anthropogenic transformation and destruction of its natural habitat continue to threaten the survival of many olive ridley turtle rookeries [1]. Furthermore, give that a turtle generation (~20 years) corresponds at least to 20 generations of the beetle *O*. *suberosus* (if we considered a generation per year, although it is possible that this species has several generations per year since the duration of its life cycle is of about 50 days only) [13] it is feasible to predict an exponential increase of beetle population since it has been reported in the last fifteen years [10]. Due to this particular scenario, predation of live and dead turtle eggs by *O*. *suberosus* has clearly been observed in the form of shell mastication and also through the presence of beetle larvae and adults in the turtle embryos [9,11,12,13] (Fig 2).

Many sea turtle populations are facing extinction and one of their main natural threats is predation of eggs and hatchlings. Predation of sea turtle embryos by opportunist beetle species has only recently been recognized [11–17]. Concretely, the beetle *O*. *suberosus* is normally necro–saprophagous in habit (adults and larvae feed on keratinose matter, mostly comprising animal materials, such as feathers, hair, skin, hair and bone in the final stages of decomposition, and carnivore droppings) [18]; however, it has also been reported as a facultative predator beetle of eggs of the turtle *L*. *olivacea* in Mexico [13], of the turtle *Chelonia mydas* (L.) in Costa Rica [14] and eggs and embryos in the Galapagos Islands (Ecuador) [16,17], even eating eggs of the grasshopper *Schistocerca cancellata* (Serville) in Argentina [19]. Furthermore, a close related species, *Omorgus procerus* (Harold), described as a predator of eggs of *Schistocerca gregaria* (Forskål) in Somalia [19], in 1966, was released on Sand Island in Honolulu to combat the invasive grasshopper *S*. *nitens* (Thunberg) [20].

A previous study confirmed that the high density of *L*. *olivacea* nests has favoured an exponential increase in the population of *O*. *suberosus*, which has been associated with a high percentage of the egg destruction at La Escobilla [13]. This study suggests that this beetle could be a considerable factor in the mortality of the embryos, thus affecting recruitment into the population of *L*. *olivacea*. The presence of *O*. *suberosus* and the proportion of damaged eggs in the nests of *L*. *olivacea* on La Escobilla is a complex phenomenon related to the dynamics of resource availability (i.e., the abundance and nutritional quality of eggs), but also to their reproductive cycle and feeding behaviour. Adult beetles are active at the surface of the beach, especially at night. However, in the turtle nests, beetles burrow down to the egg chamber where they reproduce. Beetle larvae and adults both feed on the egg contents after chewing through the shell. Beetles quickly develop to maturity and several generations overlap throughout the year [13,17]. In recent years, it has been observed that both larvae and adults of *O*. *suberosus* tend to aggregate within the turtle nests [13]. Such aggregation behaviour suggests that a subsocial factor, such as attraction to conspecifics, may be involved. This pattern is observed in many other beetle species [21–23]. On the other hand, many insect species are attracted by odours produced during protein decomposition in carrion, animal tissues and hairy or feathery animal surfaces, which are generally considered unpleasant, putrid, faecal and sulphurous (indole, skatole, putrescine, organosulphur compounds, etc.) [24–27]. Most beetles have an acute olfactory system used for locating food resources. Decomposition of biological material produces a considerable quantity of volatile organic compounds, which have been implicated in the foraging behaviour of coprophagous and necrophagous beetles [28,29]. Under natural conditions, many nests contain a combination of eggshells and live and dead eggs in an advanced state of decomposition. These volatile organic compounds emitted from the turtle nests may modulate the foraging behaviour and aggregation of *O*. *suberosus* [30]. Identification of these volatile organic compounds potentially used in food resource location is therefore important to further our understanding of *O*. *suberosus* behaviour.

The usual capture method for *O*. *suberosus* includes using traps baited with fresh chicken feathers as an attractant [13]. The use of chicken feathers as bait is economically viable; however, this method is highly labour intensive and is non-specific. Furthermore, the use of chicken (or other avian species) feathers originating from commercially farmed hens could present a health hazard for some wild bird species and could have a serious impact on human health [31]. For example, feathers are known vectors of significant human diseases such as the H5N1 and HPAI viruses [32–34]. Other options for better management of beetle density could involve the use of volatile compounds that influence beetle behaviour, such as feeding and aggregation, in order to reduce the impact of the damage caused to the *L*. *olivacea* nests.

In this context, we explore a novel ecological approach to control *O*. *suberosus*. The aims of this study were to determine the composition of volatiles emitted by beetles and other elements from turtle nests using headspace sorptive extraction (HSSE) in combination with thermal desorption (TD), coupled online to a gas chromatography–mass spectrometry (GC-MS) technique. We tested synthetic reference samples of the identified substances with electroantennograms (EAG) and olfactometers in order to evaluate their electrophysiological and behavioural activity. Finally, synthetic compounds were examined in a pilot test in the field in order to test their potential for developing a control method based on a mass trapping strategy of *O*. *suberosus*.

## Materials and methods

### Ethics statements

The field and laboratory experiments of this study were all planned in conjunction with the respective authorities. The Direction of Species of Conservation Priority of CONANP specifically provided approval for the authors to conduct the experiments in this study. Furthermore, the CONANP, under the supervision of Dr. Ninel García-Tellez, DEPC-CONANP-SEMARNAT, provided specific permission for the authors to collect *O*. *suberosus* beetles and turtle eggs, as well as its manipulation in the La Escobilla Turtle Sanctuary (CONANP/AD-S/ASE/DGOR/DEPC/067/2012; CONANP/AD-S/ASE/DGOR/DEPC/068/2012; PROCER/DGOR/01/2013).

### Biological material

All samples used in this study were collected from the ‘La Escobilla Turtle Sanctuary’ located in the municipality of Santa María Tonameca, in the state of Oaxaca, Mexico (15° 47' N; 96° 44' W). Adult *O*. *suberosus* individuals were originally collected at night and transported covered with damp vermiculite in plastic boxes (80 x 30 x 50 cm) with a 5 cm diameter mesh-covered hole for ventilation. Males and females of the same age with intact antennae and legs were identified and placed into aerated plastic boxes (60 × 40 × 40 cm) according to sex, with sterile sand as substrate. The sex of the tested insects was determined by observing their genitalia with a stereomicroscope. The plastic boxes were placed in controlled environment chambers (temperature: 28°C, relative humidity: 75%, illumination: 12 h light/day). The beetles were fed with chicken feathers every three days until use in the assays.

In order to determine whether volatiles from the turtle nests play a role in the selection of *O*. *suberosus* adults, samples were collected from several turtle nests. These samples included dead hatchlings and turtle eggs. Turtle fate eggs were categorized into three conditions: ‘fresh’ (collected during oviposition), ‘live’ (one week after oviposition) and ‘dead’. These conditions were distinguished by the odour, colour and turgidity of the eggs. After volatile collection, ‘fresh’ and ‘live’ turtle eggs were relocated in the soil under the supervision of Ninel García-Tellez to assure optimal microclimatic conditions.

### Volatile collection

A static sampling technique utilizing headspace sorptive extraction (HSSE) was used to collect volatile organic compounds released by the different elements from the turtle nests and *O*. *suberosus* beetles. The compounds were collected from the headspace of glass chambers (10 cm diameter × 15 cm long), containing either 50 female or 50 male *O*. *suberosus*. Headspace sampling was also performed in glass chambers with 4 pieces of dead hatchlings, live eggs or dead eggs for volatile collection from the turtle nest. Volatile compounds were trapped on a stir bar (0.5 mm thick × 10 mm long), commercialized under the name ‘Twister^®^’ (Gerstel GmbH & Co. KG), with a coating of polydimethylsiloxane (PDMS; sorptive extraction phase). The PDMS stir bars were pre-conditioned before use by treatment with acetonitrile (HPLC-grade) for cleaning, and conditioned at 250°C for 15 h with a 75 ml/min flow of purified helium. For extraction, the PDMS stir bar was fixed within the headspace volume by magnetic force using a neodymium disc magnet (Ø 5 mm, height 3 mm) placed inside the glass chamber containing the sample and exposed for 24 h at 32°C (simulating field conditions). Following extraction, the PDMS stir bar was removed from the glass chamber and inserted into the appropriated thermal desorption glass tube (Gerstel 187 mm length × 4 mm I.D., Gerstel GmbH & Co. KG,). The stir bar was then thermally desorbed. Control assays were performed using the same procedure, but employing an empty glass chamber. Two replicates each were performed for the control and the samples.

### Volatile compound identification

To compare chemical profiles, the headspace samples were analysed by TD–GC–MS. For this, samples were desorbed using a thermal desorption system (Gerstel TDS-2, Gerstel GmbH & Co. KG), performed for 10 min at 300°C and with a helium flow rate of 55 ml/min, connected to a gas chromatograph coupled to a mass selective detector (GC–MS). The GC–MS was carried out with an Agilent 5973MS (Agilent Technologies, CA, USA) coupled to an Agilent 6890GC (Agilent Technologies, CA, USA), equipped with a HP–5MS capillary column (30 m × 0.25 mm I.D., 0.25 μm film thickness). The carrier gas was helium (1.4 ml/min constant flow). The oven temperature was programmed for 5 min at 60°C, with a 5°C/min increase to 250°C, and then held for 5 min. The injector temperature was set at 250°C (Split mode in a 50:1 ratio). The MS transfer-line was held at 280°C and the MS quadrupole and MS source temperatures were 150°C and 230°C, respectively. Mass spectra were taken in EI mode (at 70 eV) in the range of 40–450 amu, with a scanning rate of 2.65 scans/s.

The detected peaks were identified from their retention data using MSD ChemStation software (Agilent Technologies, CA, USA) and by comparison between the obtained mass spectra and those from spectral libraries (Wiley275 and NIST/EPA/NIH). Retention indices were determined using the retention time of n-alkane standards (from C7-C40, C12-C60 μg/ml in n-hexane; Supelco 49452-U and Sigma-Aldrich ASTM^®^ D544) and compared against values in the literature using a library database (Adams 1995; Pherobase http://www.pherobase.com). Identification of the volatile compounds of interest was then confirmed with commercial standards of high purity (98%) obtained from various chemical suppliers (Fluka, Sigma, Aldrich, Avocado and Acros). To verify identity, the standards were run under the same conditions as the samples, as well as spiked into samples. Identification was considered as merely tentative when based on mass spectra data only.

### Test compounds

The selection of compounds for testing was influenced by several factors: (a) their EAG activities, (b) their physicochemical properties (volatility at ambient temperature) and (c) compounds associated with protein decomposition. The synthetic compounds tetradecane, phenol, geranyl acetone, decanoic acid, benzophenone, indole, skatole, dimethyl sulphide (DMS), dimethyl disulphide (DMDS), isovaleric acid, valeric acid, linoleic acid, oleic acid, trimethylamine (Me_3_N) and ammonia (NH_3_) were obtained from Sigma-Aldrich Co. (St Louis, MO). All of the chemicals were more than 95% pure, except for the hexane (HPLC-grade), ammonia (25% v/v aqueous solution) and Me_3_N (45% v/v aqueous solution).

To obtain concentration–response curves from both sexes for the electroantennography (EAG) recordings, compounds were tested at concentrations of 0.05, 0.1, 0.5, 1.0 and 10.0 μg/ml in hexane. Since NH_3_ and Me_3_N were insoluble in hexane, solutions were prepared in distilled water at concentrations of 0.01, 0.1 and 1.0 N. These solutions were stored at –20°C until required. Immediately prior to the experiment, 1 μl of each test solution or hexane control was adsorbed onto a filter paper strip (1 cm^2^, Whatman No. 1) and placed in a Pasteur pipette (15 cm long), which served as an odour cartridge.

On the basis of EAG activity, some of these volatile compounds were used as odour sources in the behavioural and field assays. One ml of compound was loaded onto cotton tissue packed within a polyethylene scintillation vial (4 ml) with a snap-on cap (Kimble, US, Canada). For this, the solutions were diluted in distilled water; DMDS and DMS at 10 μg/ml, NH_3_ and Me_3_N at 1N. Five hundred μg of indole packed on cotton tissue in polypropylene vials (4 ml) were also used as odour source. Dispensers were freshly prepared prior to each experiment. Empty vials with cotton tissue (100 mg) served as controls.

### Electroantennography (EAG) bioassays

Electroantennogram signals were recorded with an EAG system (Syntech, Kirchzarten, Germany) consisting of a probe connected to a high-impedance DC-amplifier (Type PRG–2), a stimulus controller (CS–55) and a data acquisition interface board (Type IDAC–02). Antennae were excised from the heads of *O*. *suberosus* using micro-scissors, inserted into small droplets of electrode gel (Spectra 360, Parker Lab, NJ, USA) and mounted individually between two metal electrodes in an antenna holder, under a purified air flow (500 ml/min). A Syntech PC-based signal processing system was used to amplify and process the EAG signals. The signals were further analysed using the EAG 2010 software (Syntech, Kirchzarten, Germany).

Stimulation tests were carried out by applying a puff of humidified pure air (2 s duration per stimulus) using a stimulus controller, through a Pasteur pipette containing a given test compound and directed over the antenna through the main branch of a glass tube (7 cm long × 5 mm diameter). Testing began once a relatively stable baseline had been established. In concentration-response experiments, stimuli were applied in ascending concentrations, whereas in the other experiments these were randomly sequenced. Control (hexane) stimuli were applied at the beginning of the experiment and after each group of 6 test stimuli. Time intervals of 30 s were left between stimuli. The test solution of a pure compound was tested on five antennae from different male and female individuals. The EAG responses were initially measured as the maximum amplitude of depolarisation in mV. The EAG amplitude was then evaluated during stimulation.

### Behavioural bioassays

To investigate the behavioural responses of *O*. *suberosus* to synthetic volatile compounds with EAG activity, we used two four-arm olfactometers. These devices were similar to the four-arm olfactometer previously described and tested by Verdú et al. [35] and consisted of one central chamber containing sterile dry vermiculite (Fine grade, Egmont Commercial Ltd, Halswell, New Zealand) as a substrate, and four arms connected to plastic containers with different odour sources. The plastic containers were designed to capture the beetles that responded to the tested odour sources. Filtered air was drawn into the plastic containers of the olfactometer through an activated charcoal filter and the central walking arena was ventilated by removing the air. To eliminate any visual cues from the satellite chambers, the central chamber and the four arms were wrapped with aluminium foil. Each chemical compound was tested alone on 20 adults of both sexes (10 males and 10 females). For this test, beetles were placed into the central chamber and confronted with a volatile compound and a control as odour sources. For each replicate, two containers with volatile compound and two empty containers (control) were randomly assigned to one of the olfactometer arms. Males and females were introduced randomly into the olfactometer with each beetle only used once. After placing the beetles in the arena, there was a 10 min interval before starting the experiment to allow the beetles to adapt to their new surroundings. The experiment duration was fixed at 12 h, which was sufficient for individuals to explore the entire experimental set-up and to select an odour source. The measured response in each test was the number of *O*. *suberosus* individuals attracted by the tested odour source. A total of 15 trials were conducted for each category. After each bioassay, the olfactometer was rotated clockwise 45° to avoid positional effects. The walking arena, arms and containers were cleaned with hexane after each experiment. All of the bioassays were conducted in a laboratory at 28°C and in darkness.

### Field experiments

**Fig 3:**
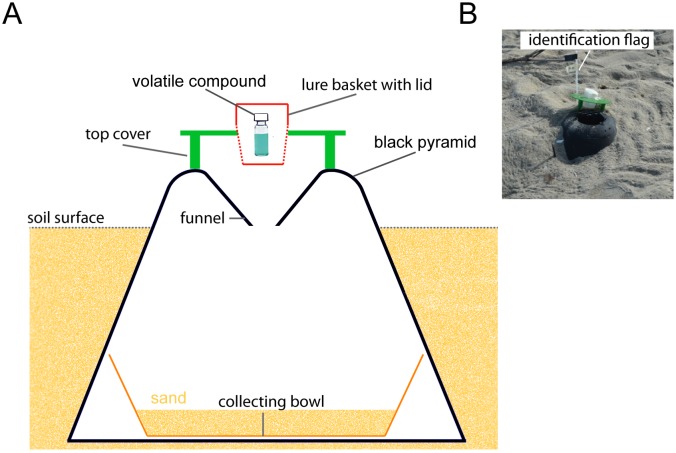
Description of the mass trapping method used for capturing *Omorgus suberosus*. A) Diagram of the standard pitfall trap AR970 (ISCA TECHNOLOGIES, USA) used and B) disposition of the trap in the field; pitfall traps were partially buried in order to reduce the distance from the edge to the entrance of the trap.

Field trials were carried out from September 26th to October 28th, 2013 (Trial 1) and from November 1st to November 5th, 2013 (Trial 2), which corresponded to the periods of oviposition of *L*. *olivacea*. All field tests took place at the turtle sanctuary of La Escobilla Beach. Maximum temperatures during the trapping period were between 28 and 32°C. Only synthetic volatile compounds that had produced results with *P* < 0.001 in the behavioural bioassays (see Results section) were selected for the field trials. Standard pitfall traps (AR970; ISCA TECHNOLOGIES, USA) were used to evaluate attraction of *O*. *suberosus*. Following Baena et al. [13], the baits (volatile compounds) were placed into the catch receptacle of each trap (Fig 3). *Omorgus suberosus* is more abundant on parts of the beach between the foreshore and the vegetation, where the highest turtle nesting activity is concentrated. In trial 1, therefore, traps were located across the width of the beach in three ecological zones: the intertidal zone (zone A), the middle of the beach (berm and platform, zone B) and within the littoral vegetation (zone C). Twenty-four sampling sites were established within each ecological zone and located at least 25 m apart. To compare the attractiveness of volatile compounds and conventional bait (chicken feathers), an empty vial with cotton tissue was used as control. Field attraction was evaluated by setting 10 traps per treatment, for a total of 70 traps. Pitfall traps were randomly distributed in each zone and installed in the ground in order to facilitate beetle access. The traps were visited every day (between 07:00 h and 14:00 h) in order to collect the beetles that had been trapped during the night. Traps were moved round every 3 days, such that each treatment was tested at each location. During sampling, the adults were removed from the traps and counted. Baits were replaced as required, depending on the prevailing temperature and relative humidity.

In Trial 2, pitfall traps baited with a combination of indole and NH_3_ were used to explore a potential chemical control strategy in the populations of *O*. *suberosus* at La Escobilla. To test whether this combination of selected compounds could increase capture rates, 30 pitfall traps were baited with indole and NH_**3**_ lures. The previous field test established the best position for the traps, and they were therefore installed within the littoral vegetation (zone C) at a distance of at least 25 m apart. After 3 nights, the pitfall traps were removed and the trapped beetles were counted.

### Statistical analyses

For analysis of the chemical compounds, the relative abundance value of the samples was calculated using the relative peak area of each compound, calculated by dividing the individual peak area by the total peak area of all compounds, and expressing the result as a percentage [36]. Similarity between the samples was calculated with a hierarchical clustering (CLUSTER) analysis using Whittaker’s index of association, which is appropriate for relative abundance data [37]. A similarity profile permutation test (SIMPROF) was used to identify statistically significant groupings within the previously obtained cluster [38]. This analysis was performed using the PRIMER 6.0 software package [37].

For the behavioural bioassays, statistic tests were conducted using the software STATISTICA v8.0 (StatSoft Inc, Tulsa, Oklahoma, USA). Prior to the analyses, we checked the normality of distribution of the dependent variable using a Kolmogorov–Smirnov test. The data were normally distributed and analysed further by using the generalised linear model (GLM) for statistical analysis. In the GLM model, two factors (compounds and concentrations) and their interactions were analysed. The GLM analysis was followed by a Tukey–Kramer HSD comparison test to determine whether there were significant differences in the beetle responses to different treatments (*P* < 0.05).

For the behaviour research, the mean number of *O*. *suberosus* obtained from the olfactometer and the scores of attractiveness test in each laboratory experiment were compared using the nonparametric Kruskal–Wallis ANOVA at *P* < 0.05. For the field study, capture rates (expressed as the mean number of beetles captured per trap per day) were used in the statistical evaluations. The effects of different baits were analysed by one-way ANOVA. A LSD multiple comparison test was used to compare mean differences between groups at *P* < 0.05.

## Results

**Fig 4:**
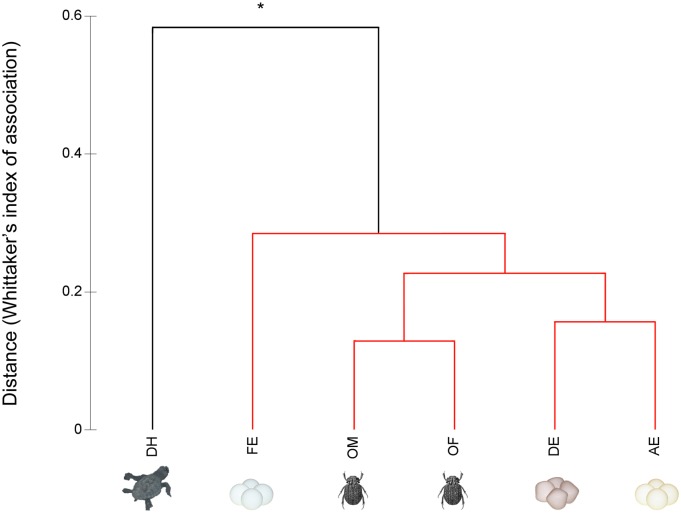
Compositional similarity analysis of volatile compounds between different food resources and secretions of *Omorgus suberosus*. Cluster and SIMPROF analysis based on Whittaker’s index of association as a distance measure of the chemical composition (DH, dead hatchlings; FE, fresh turtle eggs; LE, live turtle eggs; DE, dead turtle eggs) and beetles *Omorgus suberosus* (OM = males and OF = females). Black lines indicate significant difference (SIMPROF: *π* = 0.58; *P* < 0.05) and red lines indicate no statistically significant difference (SIMPROF: *P* > 0.05).

The HSSE–GC/MS technique determined the identities of 24 volatiles emitted from *O*. *suberosus* anal secretions and components of the *L*. *olivacea* nests (Table 1). The pattern of volatile compounds found in the samples was remarkably diverse, consisting of 11 carboxylic acids, 2 hydrocarbons (aliphatic and sesquiterpenic), 4 aromatic hydrocarbons (2 nitrogen-bearing), 4 sulphur compounds (2 polysulphides), 2 ketones (aromatic and monoterpenic) and one amine. Male and female specimens of *O*. *suberosus* contained 18 compounds while the dead *L*. *olivacea* hatchlings contained 17 compounds. Each turtle egg contained nine (live and fresh eggs) to eleven (dead eggs) identified compounds. The predominant carboxylic acids identified in this study were fatty acids. In particular, palmitic acid and linoleic acid were major components of the carboxylic acid profile. The headspace samples of dead hatchlings, dead turtle eggs and both sexes of *O*. *suberosus* contained sulphide and nitrogen-based compounds responsible for the odour of decomposition (protein decomposition). The sulphide group was heavily comprised of three oligosulphides: dimethyl sulphide (DMS), dimethyl disulphide (DMDS) and dimethyl trisulphide (DMTS). Interestingly, the nitrogen-based compounds included trimethylamine (Me_3_N), indole and 3-methylindole (also known as skatole). Cluster analysis, based on Whittaker’s index of association (Fig 4), revealed only significant chemical differences between dead turtle hatchlings and different stages of turtle eggs and *O*. *suberosus*.

**Table 1:** Profile of volatile compounds in collections from turtle nests and *Omorgus suberosus* using the HSSE/TD–GC–MS method.

Compounds	Code	RI^a^	RI^b^	Identification^c^	Relative Abundance (%)					
					*O*. *suberosus*		Dead hatchlings	Turtle eggs		
					Males	Females		Dead	Live	Fresh
**trimethylamine**	Am		329	MS; STD	2.08	1.80	9.69	n.d.	n.d.	n.d.
**dimethyl sulphide**	Sul		505	MS; STD	0.40	0.38	3.44	0.78	n.d.	n.d.
**dimethyl disulphide**	Sul		785	MS; STD	3.36	1.29	0.81	n.d.	n.d.	n.d.
isovaleric acid	Ac	828	834	MS; RI; STD	n.d.	n.d.	0.23	n.d.	n.d.	n.d.
valeric acid	Ac	910	911	MS; RI; STD	n.d.	n.d.	0.82	n.d.	n.d.	n.d.
dimethyl trisulphide	Sul	968	974	MS; RI	0.62	0.86	1.14	n.d.	n.d.	n.d.
phenol	Ar	980	980	MS; RI; STD	0.32	2.67	2.40	n.d.	n.d.	n.d.
benzothiazole	Ar	1239	1240	MS; RI; STD	0.38	0.59	n.d.	n.d.	0.36	n.d.
nonanoic acid	Ac	1278	1280	MS; RI	0.85	0.37	n.d.	n.d.	n.d.	0.34
**indole**	Ar	1290	1288	MS; RI; STD	2.05	7.49	2.35	1.54	n.d.	n.d.
caprinic acid	Ac	1373	1373	MS; RI; STD	0.69	0.60	1.79	0.30	0.35	1.10
α-copaene	Sqt	1377	1376	MS; RI	n.d.	n.d.	0.91	n.d.	n.d.	n.d.
skatole	Ar	1387	1381	MS; RI; STD	n.d.	n.d.	1.06	n.d.	n.d.	n.d.
tetradecane	Hy	1398	1400	MS; RI; STD	0.48	0.55	n.d.	n.d.	0.55	1.31
geranyl acetone	Ket	1448	1453	MS; RI; STD	2.48	0.88	n.d.	0.48	0.92	3.50
lauric acid	Ac	1572	1568	MS; RI; STD	4.09	4.49	3.59	4.66	4.44	4.02
benzophenone	Ket	1614	1604	MS; RI; STD	8.63	7.04	n.d.	n.d.	n.d.	n.d.
myristic acid	Ac	1758	1768	MS	2.12	2.92	12.77	3.13	2.38	9.51
palmitoleic acid	Ac	1947	1953	MS	n.d.	n.d.	7.74	n.d.	n.d.	n.d.
palmitic acid	Ac	1970	1984	MS; STD	32.97	29.57	15.00	37.02	51.21	46.31
sulphur compound	Sul			MS	2.28	2.17	n.d.	2.31	n.d.	n.d.
**linoleic acid**	Ac	2159	2152	MS; STD	33.01	34.74	10.92	44.15	36.84	23.70
oleic acid	Ac	2165	2161	MS; STD	3.18	n.d.	25.34	3.84	2.95	10.21
stearic acid	Ac	2174	2173	MS; STD	n.d.	1.59	n.d.	1.78	n.d.	n.d.

Compounds in bold type elicited antennal responses in the EAG experiments. Family Code: Am; Amine; Sul; Sulphur compounds; Ket: Ketones; Hy: Hydrocarbons; Ar: Aromatics; Sqt: Sesquiterpenes; Ac: carboxylic acids.

^a^ Linear retention index calculated on a DB-5 column with a homologous series of n-alkanes (C7–C30).

^b^ Linear retention on a DB-5 index in literature.

^c^ Identification proposal is indicated by the following: comparison of the MS data to Wiley 275 and NIST 02 database (MS); comparison of the Kovàts-indices literature data (RI); comparison with the retention times and mass spectra of available standards (STD). (nd) no detected.

**Fig 5:**
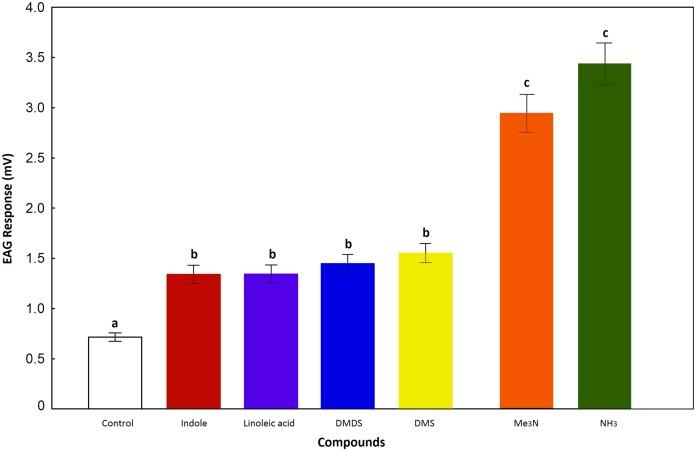
EAG-responses of *Omorgus suberosus* to volatile compounds. Vertical bars indicate standard errors. Different letters indicate significant differences in the EAG responses among volatile compounds, tested at *P* < 0.05. Hexane stimulus was used as a control.

**Fig 6:**
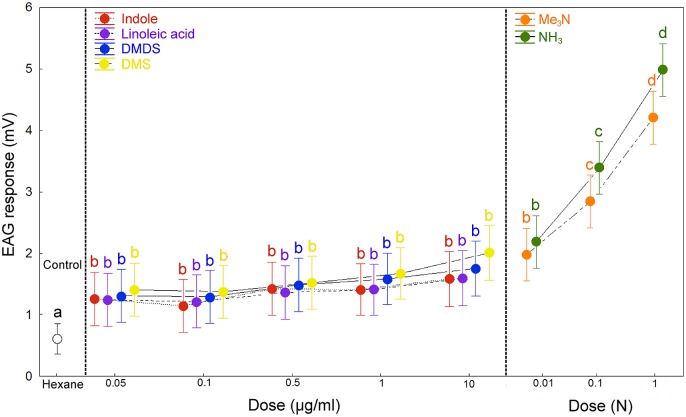
EAG concentration–response curves of *Omorgus suberosus* to different dose of chemicals. Vertical bars indicate standard errors. Different letters indicate significant differences in EAG responses among volatile compounds, tested at *P* < 0.05. Hexane stimulus was used as a control.

The EAG analysis showed that antennae of *O*. *suberosus* respond significantly to six compounds: NH_3_ (3.44 ± 0.12 mV), Me_3_N (2.94 ± 0.12mV), DMS (1.54 ± 0.16 mV), DMDS (1.44 ± 0.16 mV), indole (1.33 ± 0.16 mV) and linoleic acid (1.34 ± 0.16 mV). In general, the EAG responses differed significantly from the solvent control (hexane: 0.72 ± 0.13 mV) (Fig 5). All other chemical standards elicited responses similar to that of the solvent control and were considered as EAG-non active compounds. The compounds indole, linoleic acid, DMDS and DMS elicited similar antennal responses, which were significantly higher than that of the solvent control (*F* = 14.99; *P* < 0.0001). The tested beetles were more sensitive to Me_3_N and NH_3_ than to the other chemicals. The responses to these compounds were significantly more active than those to the solvent control (*F* = 30.51; *P* < 0.0001). Male and female EAG responses to Me_3_N were not significantly different (*F* = 1.84; *P* = 0.1762). The analysis also revealed effects of concentration on EAG response (Fig 6). However, this was not evident for all of the chemicals; only between odour stimuli Me_3_N and NH_3_ were concentration dependent (*F* = 16.13; *P* < 0.001).

**Fig 7:**
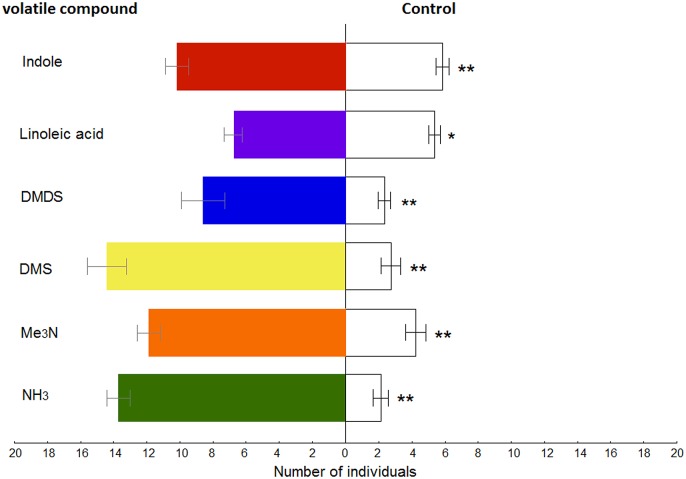
Olfactory response of *Omorgus suberosus* to active volatile compounds. Error bars represent standard errors of the mean. Statistical differences are indicated by asterisks at *P* < 0.05 (*) and *P* < 0.005 (**) respectively. An empty vial was used as control.

The behavioural responses of *O*. *suberosus* to the synthetic volatile compounds selected based on olfactory response are shown in Fig 7. The olfactometer tests showed that volatile compounds were significantly more attractive to beetles than the control: linoleic acid (KW-H_[1,34]_ = 5.3189, *P* = 0.02), DMDS (KW-H_[1,30]_ = 9.0618, *P* = 0.002), indole (KW-H_[1,30]_ = 13,8306; *P* < 0.001), Me_3_N (KW-H_[1,30]_ = 20.0647, *P* < 0.001), NH3 (KW-H_[1,30]_ = 21.6635, *P* < 0.001) and DMS (KW-H_[1,30]_ = 21.7309, *P* < 0.001).

**Fig 8:**
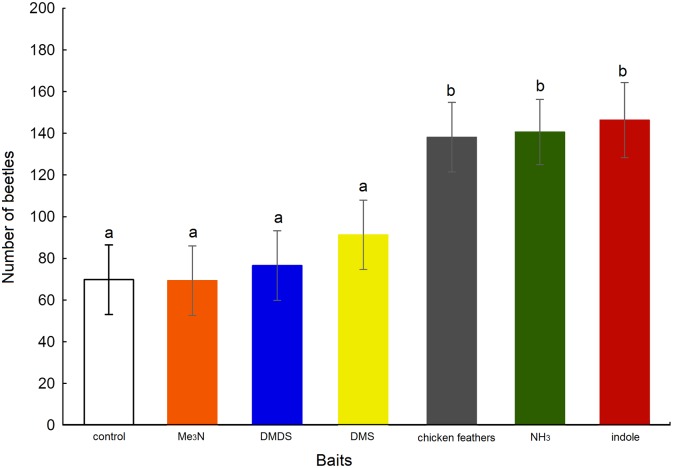
Capture rates (beetles per trap per day) of *Omorgus suberosus*, using bait. Bars indicate the means ± SE of beetles captured; same letter indicates that there was no significant difference, at a probability of 5%. An unbaited pitfall trap was used as control.

A total of 5,091 *O*. *suberosus* adults were captured in the pitfall traps. Capture rates (expressed as beetles per trap per day) of *O*. *suberosus* using different odour sources are presented in Fig 8. The analysis showed statistically significant differences between odour sources (*F*_[6,483]_ = 3.895, *P* < 0.001). Traps baited with indole, NH_3_ and chicken feathers attracted more beetles than those baited with other volatile compounds, presenting capture rates of 14.17, 13.93 and 13.81 beetles/trap, respectively, as well as trapping significantly more beetles than the control traps (capture rate = 7.10 beetles/trap; *P* < 0.01). Indole and NH_3_ were selected as the most effective baits and were used in combination for a preliminary mass trapping test for *O*. *suberosus* control. The number of beetles captured using DMS, DMDS and Me_3_N did not differ significantly from the number attracted by control traps (*P* = 0.408, *P* = 0.820 and *P* = 0.944, respectively). In the second experiment, the capture rate for traps baited with a combination of indole and NH_3_ was 17.38 beetles/trap. Based on the total of 1,564 of *O*. *suberosus* individuals captured, the traps baited with a combination of indole and NH_3_ were the most efficient of the designs tested.

## Discussion

### Compositional chemical relations and candidate semiochemicals

Following exploration of the possible volatiles emitted from all possible stages of egg maturation and decomposition, including dead turtle hatchlings, the results obtained here showed that the chemical composition of all possible odour resources emitted by eggs presents a great number of chemical compounds similar to the chemical secretions of *O*. *suberosus* (mainly anal secretions). Volatile compounds associated with protein decomposition are usually odorous for many animals, including insects [24–27]. Skatole, indole, and sulphides are the most penetrating odours of putrefaction. Another large group of attractants are fatty acids, which are usually fermentation products and decomposition components. Among all of the decomposition compounds from fats and proteins, ammonia appears to be the most common single nitrogenous product. Some of these volatile compounds are common in nature, showing diverse functions as semiochemicals. For example, trimethyalamine, indole and linoleic acid are pheromones found in the anal, urinary and facial secretions of some mammals [39–42]. Linoleic acid has been also described as a potential sex pheromone found in the skin lipids of the leopard gecko *Eublepharis macularius* (Blyth) [43]. The compounds indole and skatole have been identified as pheromones of several dung beetles of the genus *Kheper*, such as *K*. *bonellii* (Mac Leay), *K*. *lamarcki* (Mac Leay), *K*. *subanaeus* (Harold) and *K*. *nigroaeneus* (Boheman) [44–47], as well as some ant species [48,49] and the white cabbage butterfly, *Pieris rapae crucivora* (Boisduval) [50]. Organosulphur compounds, such as DMS, DMDS and DMTS, are described as attractants for the necrophagous beetles *Nicrophorus vespillo* L. and *N*. *vespilloides* Herbst (Coleoptera: Silphidae: Nicrophorinae) [24,25]. Furthermore, DMTS, DMDS and nitrogenous compounds have been identified as aggregation pheromones [50].

The results showed that all stages of the eggs studied can be considered as natural attractive to *O*. *suberosus* in the field, but also that the presence of this beetle in the nest of the turtle could act as an effect multiplier (synergetic) of the attraction and subsequently of turtle nest predation. Thus, the aggregation behaviour of *O*. *suberosus* that has been suggested by other authors [13] could be explained from this perspective, which is a very possible cause of the explosive demography of *O*. *suberosus* at La Escobilla sanctuary. Interestingly, the volatiles that characterise the chemical profiles of both the eggs and beetles contained a first group of sulphide and nitrogen-based compounds, such as DMS, DMDS, DMTS, trimethylamine, indole and skatole, that is responsible for the odour of protein decomposition, and a second group characterised by the identified carboxylic acids such as palmitic acid and linoleic acid. In other words, the chemical profile of the beetle secretions (of both sexes) was very similar to that of decomposed eggs but also of recently deposited and mature eggs, which could explain the active predation of viable eggs by *O*. *suberosus*. This surprising behaviour, which does not at all reflect the typical necro-saprophagous habit described for this beetle [18], suggests indirect predation of viable eggs rather than an active search and predation of viable eggs of *L*. *olivacea*, as is the case with *Chelonia mydas* (L.) in the Galapagos Islands [17].

### Electrophysiological and behavioural evidences

The results obtained via EAG recordings provided more clarity, since the majority of compounds that produce significant stimulus to the antennae of *O*. *suberosus* were products of protein decomposition. Among these, only trimethylamine and ammonia were concentration dependent and were the more effective semiochemicals based on the electrophysiological tests (see Figs 5 and 6). From a behavioural perspective, the olfactometer tests showed that the electrophysiological responses of antennae correspond to the significant attraction of these compounds for *O*. *suberosus*. This is the first evidence that at least six chemical compounds can be considered odour attractants for *O*. *suberosus* (Fig 7). Further investigation is required to elucidate the function of these compounds as true aggregation pheromones; however, based on these results, we suggest these six compounds as clear candidates for use in pheromone traps with which to control populations of *O*. *suberosus*.

### Preliminary field implementation

Laboratory bioassays showed a first selection of semiochemicals with potential use for mass trapping of *O*. *suberosus*. The behaviour of different chemical compounds in the field could be very diverse due to their particular chemical nature; however, these six potential volatiles were easy to manipulate and formulate in the pheromone traps. The results showed that the most effective odour attractants of *O*. *suberosus* in the field were indole (solid state) and ammonia (liquid state). This result suggests two different compounds that clearly constitute an alternative to the use of chicken feathers as bait. As stated above, the use of chicken (or other domestic birds) feathers as bait in the traps is controversial, since these can have a serious impact on human and animal health [31]. Feathers can effectively be vectors of serious human diseases such as the H5N1 and HPAI viruses [32–34]) and many other avian diseases, such as the Marek's disease virus [51], circoviral infections [52], retroviral diseases [53], and infectious laryngotracheitis (ILTV) [31], among others. Furthermore, the study area is a declared nature reserve with established populations of a great number of wild birds, some of them threatened such as the endangered Oaxaca hummingbird (*Eupherusa cyanophrys* J. S. Rowley & Orr) [54] or considered as emblematic species, such as the Emerald toucanet (*Aulacorhynchus prasinus* (Gould)), [55], the Sharp-shinned hawk (*Accipiter striatus* Vieillot) [56] and the Zone-tailed hawk (*Buteo albonotatus* Kaup) [57]. Considering this scenario, the implementation of a protocol promoted by CONANP-Mexico (which funded this research) to control the populations of *O*. *suberosus* must therefore be effective but also innocuous to the environment and in full accordance with conservation policies. Ammonia and indole are very easy and economical to obtain and manipulate, and were tested successfully in both laboratory and field experiments. Nitrogenous compounds, such as ammonia and indole are strong insect attractants. A variety of baits releasing ammonia (which is associated with protein decomposition by the action of bacteria) are used as food attractants. Ammonia bait traps are currently used for monitoring fruit fly populations (Diptera: Tephritidae) [58].

### Conclusions

In the last decade, the egg predation by the beetle *O*. *suberosus* in *L*. *olivacea* nests at La Escobilla (one of the most important beaches in the world for the reproduction of this species) has been considered a potential factor of risk to the eclosion of the turtle hatchlings. Coinciding with other studies [10,13], we assume the natural ecological role of *Omorgus suberosus* eating carcasses and preventing high levels of nitrogenous residues in the ecosystem, however, in the current scenario in which an increase about 126% of olive ridley turtle population in La Escobilla sanctuary has been reported [1], and the predictable exponential increase of *O*. *suberosus* population due to this anomalous increase of trophic resource, explain the necessity to develop a demographic control of this potential predator. It is evident that more data about temporal demographic fluctuation of both olive ridley turtle and *O*. *suberosus* are needed, but the current densities of *O*. *suberosus* observed in La Escobilla sanctuary (about 30 individuals/m^2^) [13] are sufficient to develop a preventing method of biological control of *O*. *suberosus*, Thus, in this scenario, the creation of a safe and efficient control method for preventing live egg predation is highly recommended in these situations without forgetting the important ecological role of this beetle. To our knowledge, this is the first approach involving a chemical ecology study of *O*. *suberosus*. Based on our results, the application of a mass trapping method with synthetic attractants could represent an effective measure to reduce the impact of predation. Our findings allow the timely implementation of such a trapping method for controlling populations of *O*. *suberosus* on several beaches where required.
